# The Relationship Between Height and Income With Potential Application to Treatment of Limb Length Discrepancy

**DOI:** 10.7759/cureus.56331

**Published:** 2024-03-17

**Authors:** Alexander S Rascoe, Eric J Peng, Dre'Marcus Ferrell, Jonathan A Copp, Raymond w Liu

**Affiliations:** 1 Orthopedic Surgery, University Hospitals Cleveland Medical Center, Cleveland, USA; 2 Internal Medicine, Chester County Hospital, West Chester, USA; 3 Pediatric Orthopedics, University Hospitals Cleveland Medical Center, Cleveland, USA; 4 Orthopedic Surgery, Forrest General Hospital, Hattiesburg, USA

**Keywords:** epiphyseodesis, lengthening, income, height, limb deformity, limb length discrepancy

## Abstract

Purpose

When treating limb length discrepancy (LLD), decisions regarding lengthening versus contralateral shortening require careful consideration of deformity and patient factors. Using the National Longitudinal Survey of Youth 1979 (NLSY79) database, and income as a quantitative representation of overall socioeconomic benefit, we sought to determine the height at which incremental gains in height have the greatest value.

Methods

Using the NLSY79 database, we collected demographic data, height, yearly income from wages, college education (full- or part-time), and receipt of government financial aid. Multiple-linear regression and graphical analysis were performed.

Results

The study population included 9,652 individuals, 4,775 (49.5%) males and 4,877 (50.5%) females. Mean heights were 70.0±3.0 inches and 64.3±2.6 inches for males and females, respectively. Multiple-linear regression analysis (adjusted-r²=0.33) demonstrated height had a standardized-ß=0.097 (p<0.001), even when accounting for confounding factors. Using graphical analysis, we estimated cut-offs of 74 inches for males and 69 inches for females, beyond which income decreased with incremental height.

Conclusions

Using income as a quantitative representation of socioeconomic value, our analysis found income increased with incremental height in individuals with predicted heights up to 74 inches for males and 69 inches for females. Shortening procedures might receive more consideration at predicted heights greater than these cut-offs, while lengthening might be more strongly considered at the lower ranges of height. Additionally, our multiple-linear regression analysis confirms the correlation between height and income, when factoring in other predictors of income.

## Introduction

In the treatment of limb-length discrepancy (LLD), orthopaedic surgeons play a pivotal role in steering a patient’s ultimate height through a choice of either limb-lengthening, limb-shortening, or combination surgical procedures. The choice depends on many patient-dependent factors including the characteristics of the deformity, the expected height remaining, patient preference, and the patient’s ability to comply with the outlined treatment plan. 

Pediatric orthopaedic surgeons have favored epiphysiodesis in children with LLD ranging from 2-5 cm (~0.78 to 2 inches) while preferring limb lengthening when >5 cm [1,2]. However, lengthening may be preferred if there is an additional deformity in the short limb to concurrently correct. Shortening of mild LLD with epiphysiodesis is thought to minimally weaken the musculature, and the loss of ultimate height is deemed a reasonable trade-off for avoiding a larger reconstructive procedure [1].

The advancing technology of internal lengthening nails has expanded the clinical options for addressing LLD [3]. This implant may change the balance between shortening and lengthening approaches for the 2-5 cm range, by offering a decreased morbidity of lengthening. These implants have been utilized in patients with LLD greater than 2 cm with acceptable complication rates [4].

While the orthopaedic literature remains undecided regarding the treatment algorithm for LLD, the importance of ultimate height is significant. Numerous studies have associated height with various human behaviors. Taller individuals have shown lower rates of suicide, and are more likely to report positive than negative emotions [5,6]. Increased height has also been positively correlated with individual happiness in a well-being study conducted in the Italian population [7]. Marriage rates in India have also been shown to correlate with height, with taller females more likely to marry than shorter females [8]. Shorter maternal height has even been associated with a higher risk of pre-term birth in a study of Swedish females [9]. 

Given these established associations, defining the threshold for diminishing gains in socioeconomic value from incremental height increases is important. Understanding the difficulty of quantifying and analyzing the full spectrum of factors known to be associated with height, we chose to evaluate income from wages as a quantitative marker of an individual’s societal success [5-13]. Using the National Longitudinal Survey of Youth 1979 (NLSY79) database we aimed to quantify the nature of the relationship between height and income while controlling for factors that may change income expectations.

## Materials and methods

Subjects

The NLSY79 database is drawn from a nationally representative Bureau of Labor Statistics survey beginning in 1979, of 14- to 22-year-old American youths [14]. Due to the universal public accessibility of this database, institutional review board (IRB) approval was not required. The original survey was constructed of 12,686 subjects with three subsamples. One was intended to be cross-sectional of the United States population, comprising 6,111 individuals, another to oversample economically disadvantaged youths (n=5,295), and a military sample (n=1,280). For administrative reasons, the survey of the non-Black and non-Hispanic (survey-defined categorization which we have maintained for transparency) portion of the economically disadvantaged subsample was stopped, as was the majority (n=1,079 of 1,280) of the military sample.

Methods

**Figure 1:**
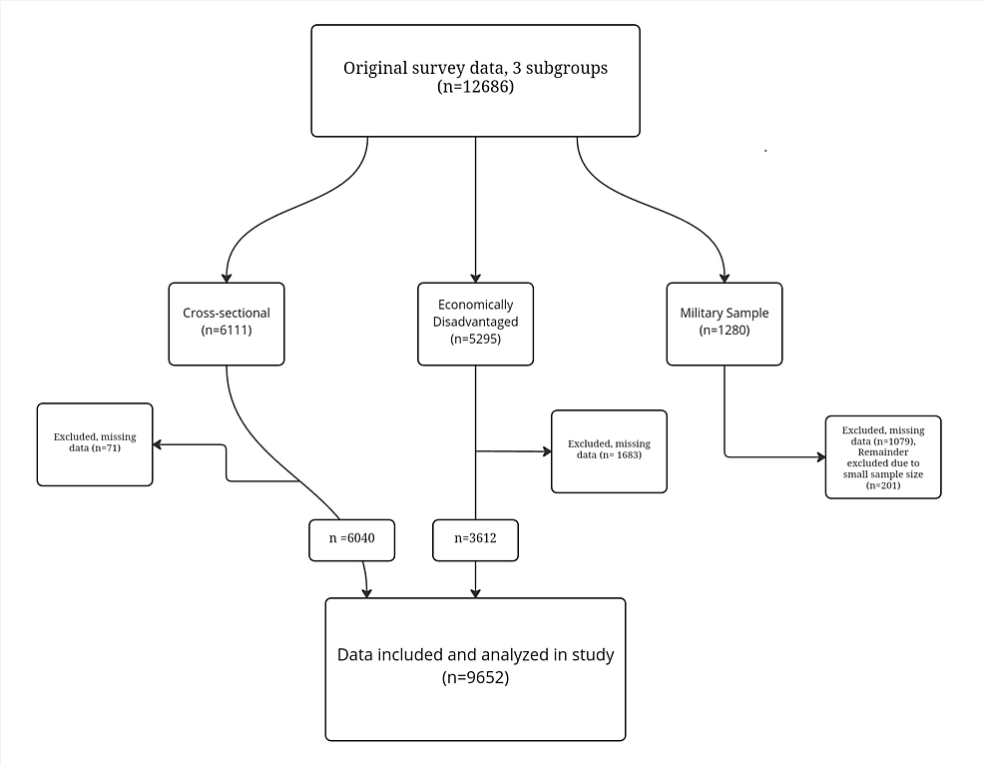
Subject cohorts from original survey vs. included in study analysis. Flowchart depicting the three subsamples: Cross-sectional, economically disadvantaged, and a military sample taken from the original survey data (n=12,686) vs. survey data included in study analysis (n=9,652)

We selected cases from the cross-sectional subsample and the economically disadvantaged youth subsample. Individuals with incomplete data as noted above, or not reporting height were excluded resulting in a cross-sectional subsample (n=6,040) and an economically disadvantaged subsample of Hispanic and Black individuals (n=3,612). The remainder of the military sample was excluded due to the small remaining sample size (Figure 1). We created a third group by pooling cross-sectional and economically disadvantaged into one subsample, to evaluate the full spectrum of cases, the combined subsample. For each of these groups, we collected demographic information regarding subject sex, age as of 1983, and race (Hispanic, Black, Caucasian, Asian, and other ethnicities). We calculated average height and yearly income from wages from 1983-2000.

We further recorded if these individuals had participated in any form of college education (full- or part-time) and/or had received any form of government financial aid. Before analysis, income from wages was adjusted to 2016 US dollars by use of a gross domestic product (GDP) deflator [15]. Average income was tabulated across the duration of the study period for everyone. The number of years an individual completed self-reported income data was tabulated.

Statistical analysis

Multiple-linear regression analysis was performed to determine the importance of factors predictive of average income from wages with Statistical Package for Social Sciences (SPSS; IBM Corp., Armonk, NY, USA). Constant values were included in model creation but are not reported. Additionally, we undertook a graphical methodology of analyzing the relationship between height and income in the combined subsample by determining the average income at each height level for which five or more individuals reported height. We then graphically fit the data with linear, quadratic, exponential, logarithmic, and power functions to aid in quantifying the trends observed.

## Results

The cross-sectional subsample consisted of 6,040 individuals, of whom 2,966 (49%) were male and 3,074 (51%) were female (Table 1). Heights ranged from 55-83 inches and averaged 70.3 inches for males and 64.5 inches for females. The average male income from wages was $33,437 and the average female income from wages was $19,262. 

**Table 1:** Descriptive characteristics of cross-sectional subsample.

Cross-Sectional, n=6,040	Male	Female
Average Age of 1983 in Years (Average±σ)	22.3±2.2	22.4±2.2
Sex (% of sample)	2966 (49.1%)	3074 (50.8%)
Height in Inches (Average±σ)	70.3±2.8	64.5±2.6
Income in US Dollars	$33,437	$19,263
College Attendance (% of sex)	1639 (55.3%)	1935 (63.0%)
Receipt of Government Financial Aid (% of sex)	793 (26.7%)	1154 (37.5%)

The economically disadvantaged subsample consisted of 3,612 individuals, of whom 1,809 (50%) were male and 1,803 (50%) were female (Table 2). Heights ranged from 53-70 inches and averaged 69.4 for males and 63.9 for females. The average male income from wages in the combined subsample was $23,358 and the average female income from wages was $15,780. 

**Table 2:** Descriptive characteristics of economically disadvantaged subsample

Economically Disadvantaged, n=3,612	Male	Female
Average Age of 1983 in Years (Average±σ)	22.2±2.2	22.4±2.2
Sex (% of sample)	1809 (49.9%)	1803 (50.1%)
Height in Inches (Average±σ)	69.4±3.1	63.8±2.7
Income in US Dollars	$23,358	$15,780
College Attendance (% of sex)	807 (44.6%)	1059 (58.7%)
Receipt of Government Financial Aid (% of sex)	728 (40.2%)	1059 (58.7%)

The combined subsample consisted of 9,652 individuals with heights ranging from 53-83 inches and other demographic characteristics as summarized in Table 3.

**Table 3:** Descriptive characteristics of combined subsample.

Combined Subsamples, n=9,652	Male	Female
Average Age of 1983 in Years (Average±σ)	22.3±2.2	22.4±2.2
Sex (n)	4775 (49.5%)	4877 (50.5%)
Height in Inches (Average±σ)	70.0±3.0	64.3±2.6
Income in US Dollars	$29,619	$17,975
College Attendance (n)	2,446 (51.2%)	2,994 (61.4%)
Receipt of Government Financial Aid (n)	1521 (31.9%)	2213 (45.4%)

The multi-linear regression model constructed for the combined subsamples reached statistical significance (p<0.001), as did the following parameters: sex, subject age as of 1983, height, receipt of government financial aid, number of years wages from income were reported, Hispanic race, non-Black and Hispanic race, and college attendance (adjusted-Pearson correlation coefficient = 0.327). 

**Figure 2:**
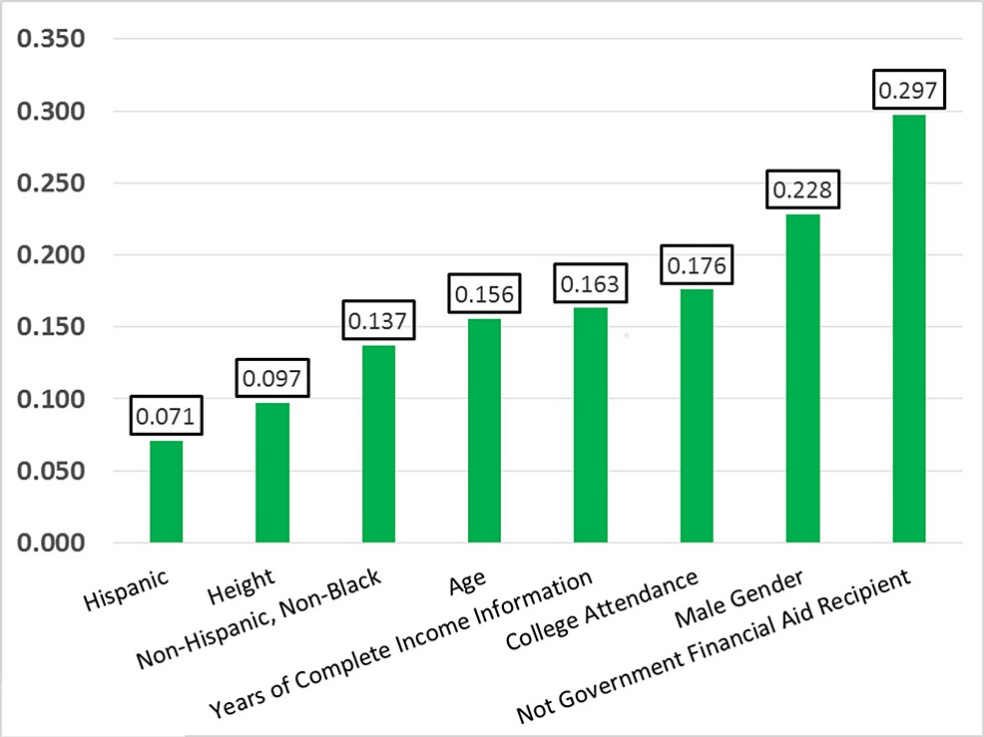
Standardized Beta Coefficients from Multiple-Linear Regression Model. Standardized coefficient values reaching statistical significance (p<0.05) from the multiple-linear regression model predicting average income within the combined subsample (n=9,652). The most significant factor predictive of income in this model was not having received government financial aid, the height term reached statistical significance with a beta value of 0.097

For each additional inch of height, average annual income from wages increased by $466.20, or 1.8% of the combined subsamples average yearly income from wages. The most predictive factors of income from wages were having not received government financial aid, male sex, and having attended college (full- or part-time) (Figure 2). The values for the standardized beta coefficients for the cross-section and economically disadvantaged subsamples are enumerated in Table 4.

**Table 4:** Multiple-Linear Regression Standardized Coefficients for Cross-Sectional and Economically Disadvantaged subsamples.

Cross-Sectional	Adjusted-r^2^			Economically Disadvantaged	Adjusted-r^2^	
	0.318				0.306	
Factor	β Coefficient	p-value		Factor	β Coefficient	p-value
Not Government Financial Aid Recipient	0.282	< .001		Not Government Financial Aid Recipient	0.350	< .001
Male Sex	0.268	< .001		Male Sex	0.158	< .001
College	0.162	< .001		College	0.202	< .001
Years of Completed Income	0.158	< .001		Years of Completed Income	0.183	< .001
Age	0.159	< .001		Age	0.156	< .001
Non-Hispanic, Non-black	0.081	< .001		Non-Hispanic, Non-black	n/a	n/a
Height	0.100	< .001		Height	0.091	< .001
Hispanic	0.049	< .001		Black	-0.095	< .001

**Figure 3:**
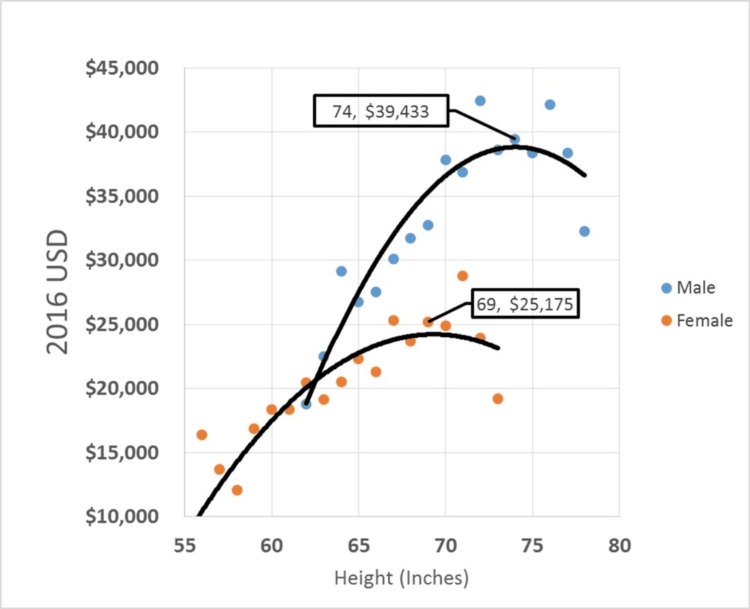
Average Income at Each Height Level. Graphical representation of average income reported at each height level for which five or more individuals reported income within the combined subsample (Table 3). Trend lines from the best fit quadratic functions are shown with maximal values for income determined at 74 inches for men and 69 inches for women

We also graphically report the average income, at each height level, for which five or more individuals reported that height (Figure 3). We then fit various functions described in the methods and graphically found that quadratic equations best fit our scatterplots, with maximum points at 74 inches for males and 69 inches for females. Beyond these values, income decreased with increasing height.

## Discussion

Using income as a surrogate for socioeconomic value, our study investigated the relationship between height and income in a large prospectively collected government-organized and funded database, while controlling for multiple factors influencing income. The graphical maximal values for average income were obtained at 74 inches for males and 69 inches for females (Figure 3). These values likely represent diminishing gains and then reversal of the positive correlation between height and income from wages. The novelty of this analysis lies in our focus on the incremental benefit of ultimate height, which is relevant when addressing LLD. Although many surgeons might consider varying their treatment strategies for LLD in patients with more dramatically short or tall predicted ultimate heights (e.g. under 60 inches, above 75 inches), this is the first study to our knowledge that offers data that may aid in selecting a limb shortening versus lengthening approach for patients with LLD in the 2-5 cm range.

Our results are consistent with a similar analysis performed by Judge and Cable, also using the NLSY79 database which provides an external validation of our methodology. These authors found a stronger standardized beta of height predicting income of 0.20, compared to our value of 0.097 [16]. Their methodology had several key differences from ours; first, they included only 4,314 subjects and only included gender, age, weight, and height in their multi-linear regression model with an overall Pearson correlation coefficient of 0.13. Comparatively, our model included factors for college participation, receipt of financial aid, race, and the number of times an individual self-reported income, yielding a larger overall Pearson correlation coefficient of 0.327 in the combined subsample. Regardless of methodological differences, a significant association between height and income in this dataset was observed by two separate, independent analyses.

There are important limitations to consider while interpreting the results of this study. Firstly, the database is over 40 years old and may not account for advancements in pre-natal, in-utero, and nutritional support. Also, while we consider nutritional support via food stamps, we have not standardized participants’ baseline nutritional status. Lastly, our outcome variable, wages from income, is based on self-reports which may not capture additional sources of revenue. However, given the substantial number of participants included in the survey, it is unlikely there is a large discrepancy in the trends for income from wages versus total wealth.

It is important to note that we are not implying a causative association between increased height and increased income. Instead, we acknowledge a correlation between the two which is independent of several important factors including education, the typical pathway to obtaining greater earnings. We therefore considered how such a relationship could exist in our society. One recent study tested the relationship between height and individual self-perception, by using virtual reality which augmented subjects’ height as compared to their surroundings. The results demonstrated that by decreasing relative height, subjects experienced diminished self-comparison (self-evaluation relative to peers) and increased propensity for paranoid behavior [17]. The association between mood disturbances and height appears to disproportionately affect those with shorter stature. A separate study of 1,299,177 Swedish males born between 1950-1981 has demonstrated a 9% decrease in suicide risk for every 5 cm height increase [5]. 

Furthermore, evidence exists that taller individuals are perceived by others differently. A study in the Dutch population determined that increased height changed male and female leadership perceptions. By displaying photographs in an online survey-response format, the authors found that photographs altered to make the individuals appear taller were more likely to have leadership qualities attributed, with a stronger effect for males than females [18]. These studies suggest taller individuals may have a propensity for being granted leadership roles which tend to earn higher pay, with evidence showing taller individuals were more likely to select higher-paying jobs [12]. Elaborating the precise relationship between height and income remains out of the purview of the orthopaedic surgery literature; however, establishing the understanding of a leveling out of the association between height and income may help determine LLD treatment strategies. 

In many clinical situations, decisions to equalize LLD occur in the juvenile to adolescent age range. One might argue that the advantages conferred by height may have already affected individuals before this time. Indeed, the authors of the suicide risk study above speculated that shorter individuals may experience increased discrimination as children, a finding consistent with a separate study of bullying in the English school system [13]. In this cross-sectional study, shorter children reported being bullied and spending their break time alone more frequently than their taller counterparts. These early years and experiences in a child’s life are formative, and it is a major limitation of our study that it is difficult to differentiate when, in an individual’s lifetime, height may be most influential. Still, we suspect that the timing is likely earlier as opposed to later and continues throughout development. 

Since orthopaedic surgeons already have tools to predict the ultimate height of a growing child, they may consider whether that child will ultimately fall above or below the “shorter” or “taller” parameters established in this study [19,20]. In this way, ultimate height could become an important factor when evaluating treatment options for LLD.

## Conclusions

The results presented in this study confirm a correlation between height and a robust measure of societal success, namely wages from income, even when accounting for gender, age, race, educational attainment and a proxy for socioeconomic status, receipt of government aid. These results offer important considerations for surgeons treating LLD with height preservation procedures including internal-lengthening nails or hexapod frame application versus a more conservative epiphyseodesis, and to our knowledge is novel to the orthopaedic literature. Future research studies should consider ultimate height when evaluating the benefits of limb equalization surgery.
